# Investigation of genetic polymorphisms in genes encoding growth factors and dental pulp calcification in orthodontic patients

**DOI:** 10.1016/j.jobcr.2024.09.013

**Published:** 2024-10-02

**Authors:** Sandra Regina Santos Meyfarth, Iago Ramirez, Alice Corrêa Silva-Sousa, Peter Proff, Marilisa Carneiro Leão Gabardo, Manoel Damião Sousa-Neto, Flares Baratto-Filho, Erika Calvano Küchler, Leonardo Santos Antunes, Christian Kirschneck

**Affiliations:** a Postgraduation Program, School of Dentistry, Fluminense Federal University, Niterói, RJ, Brazil; b School of Dentistry, University of São Paulo, Ribeirão Preto, SP, Brazil; c Department of Orthodontics, University of Regensburg, Regensburg, Germany; d School of Dentistry, Positivo University, Curitiba, PR, Brazil; e School of Dentistry, Tuiuti University from Paraná, Curitiba, PR, Brazil; f Department of Dentistry, University of Joinville Region (Univille), Joinville, SC, Brazil; g Department of Specific Formation, Fluminense Federal University, Niterói, RJ, Brazil; h Department of Orthodontics, University Hospital Bonn, Medical Faculty, Bonn, Germany

**Keywords:** Dental pulp calcification, Genetic polymorphism, EGF, EGFR, TGFβ1, TGFβR2

## Abstract

**Background and objectives:**

Pulp calcification is associated with many factors and triggers, including individual genetic predisposition and orthodontic forces. This study aimed to investigate whether genetic polymorphisms in *epidermal growth factor (EGF), epidermal growth factor receptor (EGFR1), transforming growth factor-beta 1 (TGFβ1), and transforming growth factor-beta receptor 2 (TGFβR2)* are associated with a risk of dental pulp calcifications in orthodontic patients.

**Materials and methods:**

Digital orthopantomography (OPG) and genomic DNA from 132 patients were analyzed in this cross-sectional study. Pulp calcification was observed in the maxillary and mandibular first molars. Genomic DNA extracted from saliva cells was used to genotype eight genetic polymorphisms using real-time polymerase chain reaction: *EGF* (rs2237051 and rs4444903), *EGFR* (rs2227983 and rs763317), *TGFβ1* (rs1800469 and rs4803455), and *TGFβR2* (rs3087465 and rs764522). The association between pulp calcification and genetic polymorphisms was analyzed using allelic and genotypic distributions, and haplotype frequencies (*P* < 0.05).

**Results:**

The prevalence of pulp calcification was 42.4 % in 490 molars. Genotypic analysis and allelic distribution showed no statistically significant association between the evaluated growth factors and molar calcification (*P* > 0.05). No haplotype combinations showed a statistically significant difference (*P* > 0.05).

**Conclusion:**

The genetic polymorphisms investigated were not associated with dental pulp calcification in orthodontic patients. Further studies should investigate other polymorphisms in genes encoding growth factors.

## Graphical abstract

**Figure:**
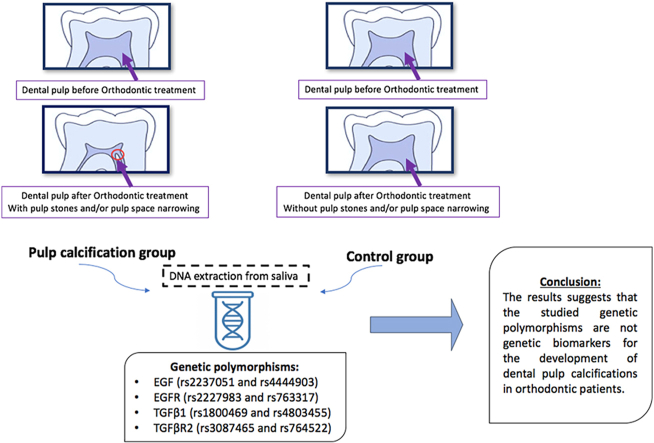

## 1 Introduction

The dental pulp is a complex and specialized non-mineralized connective tissue surrounded by a hard tissue of dentin.^1^ Dentin combined with dental pulp, denominated as the dentin–pulp complex, is capable of inducing repair under aggression.^2^ Partial or complete pulpal calcification is one of the ways in which the dentin-pulp complex is repaired under damage.^2^ Dental pulp cells differentiate into odontoblasts and produce a mineralized matrix. The reparative process occurs when there is no infection or when there is adequate blood supply, in conjunction with the participation of proinflammatory cytokines, growth factors, extracellular matrix components, and other biologically active molecules.^3^ There are two main morphological types of calcifications: discrete pulp stones (i.e., denticles or pulp nodules) and diffuse calcifications.^4^

The prevalence of pulp calcification (8–95 %) varies^5^, ^6^, ^7^ depending on the population, study type and design, and evaluation method.^8^ However, at least 50 % of all teeth have one or more calcifications, mostly in the molars.^9^ Although the etiology of pulp calcification is unknown,^8^ it has been associated with some predisposing factors, including age,^10^ trauma,^11^ angiogenic factors such as poor circulatory supply,^12^ orthodontic movements,^13^^,^^14^ and some genetics factors (patient genetic background).^15^^,^^16^

A recent systematic review showed that orthodontic forces trigger a sequence of biological responses that accelerate pulp tissue calcification metabolism, which may increase the incidence of pulp calcification.^13^ Another study has reported that when orthodontic forces are applied to the teeth over a period of time, the expression of various growth factors in pulp tissues increases, including epidermal growth factor (EGF) and transforming growth factor-beta (TGFβ).^17^ The authors concluded that the growth factors, assessed in their study, released following orthodontic force application play an important role in the angiogenic response of the pulp,^17^ suggesting that the genes encoding EGF and TGFβ family members could be involved in dental pulp phenotypes.

Dentin formation is well-known controlled by several genetic factors, including growth factors.^18^ The dentin matrix contains several growth factors with potent biological effects; after secretion, they interact with other components of the extracellular matrix, thus becoming incorporated into the dentin matrix.^19^ The bioactive components of the dentin matrix play crucial roles in pulp tissue healing and repair.^20^ The TGFβ family constitutes three isoforms (TGFβ1, TGFβ2, and TGFβ3) and has a central role in tooth development morphogenesis and odontoblastic differentiation. More specifically, TGFβ induces the secretion of extracellular matrix and pulpal calcification, especially in pulp-dentin pathophysiology state.^21^^,^^22^ TGFβ binds to the type II TGFβ receptor (TGFβR2). TGFβ signaling is principally mediated through its receptors, which are expressed in both odontoblasts and pulp cells with TGFβ isoforms.^23^ EGF is a unique polypeptide involved in the regulation of cell proliferation that exerts its effects on target cells by binding to the EGF receptor (EGFR) located on the plasma membrane.^24^ Moreover, EGF is a mitogen for fibroblasts and endothelial cells *in vitro*, promoting angiogenesis *in vivo*.^25^ Derringer et al.^17^ found that after the application of orthodontic force, EGF promoted angiogenic changes in the human dental pulp.

Dental pulp calcifications have a strong genetic background.^15^^,^^16^^,^^26^ A recent study showed that genetic polymorphisms in *WNT10A* and *WNT6* may be considered biomarkers of dental pulp calcifications in orthodontic patients.^16^ WNT10A and WNT6 cooperates with TGFβ to regulate odontogenic differentiation^27^ suggesting that genetic polymorphisms in growth factors could also be associated with dental pulp calcification. Therefore, considering the known functions of growth factors, this research aimed to investigate whether there is an association between well-investigated genetic polymorphisms in the literature in *EGF* (rs2237051 and rs4444903), *EGFR* (rs2227983 and rs763317), *TGFβ1* (rs1800469 and rs4803455), and *TGFβR2* (rs3087465 and rs764522), and to elucidate the risk of dental pulp calcifications in orthodontic patients. The null hypothesis was that there was no association between the selected genetic polymorphisms and dental pulp calcifications.

## 2 Material and methods

### 2.1 Ethical aspects and study design

This study has been approved by the Research Ethics Committee of Regensburg University (approval number: 19-1549-101). The study was conducted in accordance with the latest version of the Declaration of Helsinki. All patients were informed about the study and written consent was obtained from legal guardians and/or participants. This genetic cohort study followed the Strengthening the Reporting of Genetic Association Study (STREGA) Statement Checklist.^28^

### 2.2 Inclusion/exclusion criteria

This study included subjects who had undergone previous orthodontic treatment, with no extraction of the upper and lower first and second premolars and molars, with no tooth loss due to carious lesions or trauma, without agenesis of permanent teeth (with the exception of the third molars), and without severe malocclusion.

Patients with systemic condition and a positive history of medical disorder such as syndromes, or oral cleft were excluded. Molars presenting with deep carious lesions or dental restorations/fillings, prosthetic dental crowns, root canal/endodontic treatment, teeth with open apexes, developmental dental anomalies compromising radiographic evaluation, previous history of dento-facial trauma,^29^ and pulp calcification identified prior to orthodontic treatment were also excluded from the study. Low-quality radiographs (such as incorrect angulation, inadequate exposure, or faulty processing) were not analyzed.

Orthodontic variables that could influence the pulp calcification outcome, such as age at the start and end of treatment, orthodontic treatment duration (in years), and the type of facial profile (sagittal skeletal malocclusion), were also considered and defined according to the ANB Steiner angle as follows: Class 1 (0^o^< ANB<4^o^), Class 2 (ANB≥4°), and Class 3 (ANB≤0°).

### 2.3 Samples description

GPower software (Franz Faul University, Kiel, Germany) was used for sample calculation assuming a prevalence of dental pulp calcification in the first molars similar to 50 %,^30^ with α = 0.05 and β = 0.20 as parameters. Based on the average alleles frequencies of the studied genetic polymorphisms, and expecting a 25 % difference between groups, a minimum of 120 patients were required.

**Fig. 1:**
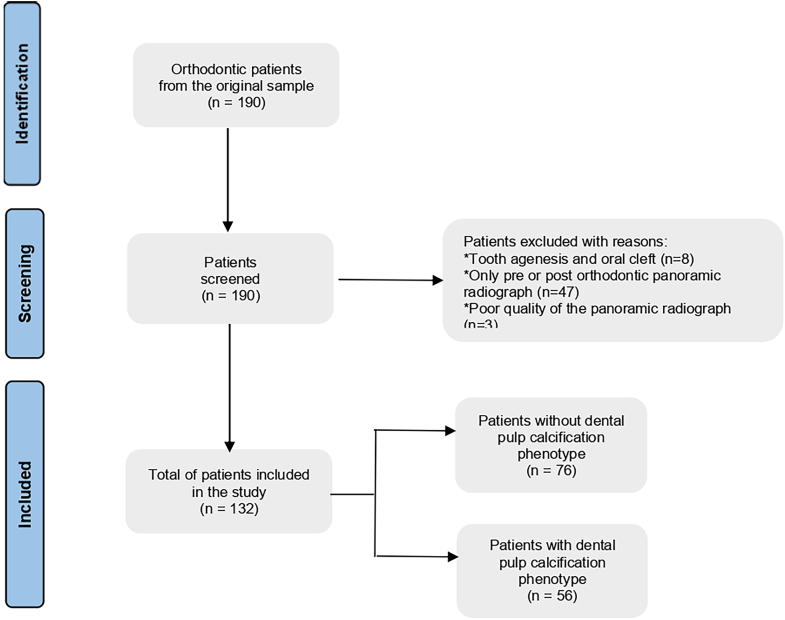
Flowchart of the study according to STREGA statement checklist to the sample selection.

The samples were briefly described in our previous study^16^: initially, 190 patients (aged ranging from 7 to 18 years) who underwent orthodontic treatment with fixed appliances at Regensburg University were selected. Pre- and post-orthodontic digital orthopantomography (OPG) and genomic DNA from these patients were assessed. All maxillary and mandibular permanent first molars were evaluated for partial or complete pulp calcification. These teeth were chosen because of their anchoring role during orthodontic treatment.^16^ After applying the inclusion and exclusion criteria, 490 first molars from 132 patients (71 females, 61 males) were analyzed (Fig. 1). The mean age of the patients at the beginning of treatment was 14.6 (standard deviation = 2.4) and the mean age at the end of treatment was 16.4 (standard deviation = 2.2).

Thirty-eight patients had 3 first molars included and 94 patients had all 4 first molars included.

### 2.4 Evaluation of dental pulp calcification phenotype

Dental pulp calcification was analyzed by two endodontists. Any disagreement was resolved through discussion until an agreement was reached. Cohen's kappa was used for inter-(0.81) and intra-observer (0.94) consensus. All radiographs that met the high-quality requirements, such as correct angulation, proper exposure, and adequate processing, were examined digitally in a dark room^29^ using Windows Photo Viewer software for Windows 10 (Microsoft Corporation, Redmond, WA, USA) on a 14-in Lenovo 81V7S00100 monitor (Lenovo PC International, Beijing, China) with a resolution of 1360 × 768 pixels. When necessary, the endodontists manipulated the images contrast and brightness to improve their quality and obtain the clearest images in the analyzed areas.^31^

**Fig. 2:**
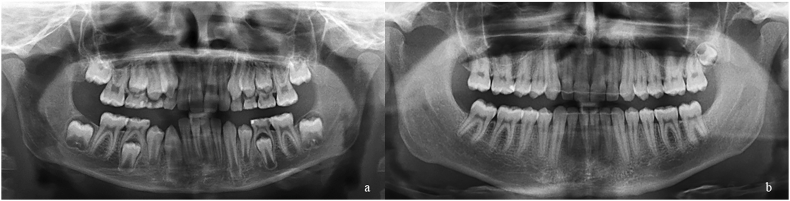
Panoramic radiographs before and after orthodontic treatment. a) pre-orthodontic treatment panoramic radiograph; b) post-orthodontic treatment panoramic radiograph showing first upper and lower molars presenting narrowing of the pulp space.

Pulp calcification in the molar tooth was recorded when calcified pulpal masses were radiographically observed in the pulp chamber and/or root canal. To determine pulp calcification, the first molar should present with pulp stones in the coronal and/or radicular parts of the dental pulp cavity and/or narrowing of the pulp space in the chambers and/or root canals. Pulp stones are round, oval, or irregular radiopaque masses within the dental pulp chamber, embedded in or attached to dentin.^32^ In contrast, the narrowing of the pulp space results in a considerable reduction in the volume of the pulp chamber and/or root canals.^31^ Based on these radiographic parameters, the molars were assessed for presence or absence of pulp calcifications after orthodontic treatment (Fig. 2).

### 2.5 Selection of the studied genetic polymorphisms

Genes selection was based on previous published studies, suggesting that these genes are involved in the dental pulp calcification phenotype. TGFβ plays a key role in dental tissue morphogenesis and odontoblastic differentiation. Additionally, TGFβ induces the secretion of extracellular matrix and pulpal calcification, especially in pulp-dentin pathophysiology state.^21^^,^^22^ EGF is a potent mitogen for fibroblasts and endothelial cells that promotes angiogenic changes in human dental pulp.^25^

A total of 8 genetic polymorphisms (two in *TGFβ-1*, two in *TGFβR-2*, two in *EGF*, and two in *EGFR*) were selected based on their clinical relevance observed in previous studies, or for their ability to influence gene expression by increasing or decreasing the final amount of protein encoded by each gene, or when the polymorphism produces amino acid change in the encoding protein. These aspects of each polymorphism are described in Table 1. Also, only genetic polymorphisms with minor allele frequency higher than 20 % were screened.

**Table 1:** Characteristics of the studied genetic polymorphisms.

Genes	Chromosome	rs	Base change^a^	Minor Allele Frequency^b^	Description
***EGF***	4:109912954	rs4444903	A > G	0.469	Located in the *EGF* 5′untranslated region, contains a base change of Guanine with an Adenine. It is one of the most important polymorphisms in the *EGF* gene; enhance mineralization during differentiation of mesenchymal stem cells derived from bone marrow.^51^
	4:109980042	rs2237051	G > A	0.412	Non-synonymous polymorphism in the coding region of the EGF gene that causes a change from isoleucine to methionine at amino acid position 708. It was related to several types of tumors, inflammatory diseases, and generalized aggressive periodontitis.^47^
***EGFR***	7:55161562	rs2227983	G > A	0.223	Includes a Guanine with an Adenine transition leading to an Arginine to Lysine substitution at amino acid position 521. It has been associated with tumors.^53^
	7:55027504	rs763317	A > G	0.395	Located in the first intron of the *EGFR* gene; it has been associated with tumors.^52^
***TGFβ1***	19:41354391	rs1800469	A > G	0.327	Located in the promoter region of the *TGFβ1* gene. It has the function of regulating expression levels of protein *TGFβ1*(affects gene transcriptional activity and serum levels of *TGFβ1*).^39^
	19:41345604	rs4803455	C > A	0.480	Located in intron 2 of *TGFβ1* gene and was associated with a variety of different conditions.^39^
***TGFβR2***	3:30605668	rs3087465	A > G	0.371	It is located in the promoter region of the gene and increases *TGFβ* type II receptor expression.^39^
	3:30605058	rs764522	G > C	0.245	It is located in 5 kb upstream in the promoter region of *TGFβR2* increases TGFβ type II receptor expression.^39^

Note: data also obtained from databases.

a http://www.thermofisher.com.

b https://www.ncbi.nlm.nih.gov/snp.

The characteristics of the selected genetic polymorphisms, such as minor allele frequency according to dbSNP, chromosome location and base change, are shown in Table 1.

### 2.6 DNA extraction and genotyping analysis

Initially, saliva samples were collected from all participants. For genotyping analysis, the genomic DNA was extracted from buccal cells isolated from the saliva using an established protocol by Küchler et al.^33^ Spectrophotometry (Nanodrop 1000, Thermo Scientific, Wilmington, DE, USA) with 2 μL of extracted material was used to determine the concentration and purity of the DNA. Only DNA samples with the ratio of absorbance at 260 nm and 280 nm with a ratio lower than 1.8 were used.

The Taqman™ method for real-time polymerase chain reaction (PCR) in the Mastercycler®ep realplex-S thermocycler, Eppendorf AG (Hamburg, Germany) was used to genotype the selected genes. All the genotype procedures were blindly performed in a total volume of 3 μL: 4 ng DNA/reaction, 1.5 μL Taqman PCR master mix, and 0.1 μL SNP assay (Applied Biosystems, Foster City, CA, USA) and, deionized water. For thermal cycling it was set an initial hold cycle of 95 °C for 10 min, followed by 40 amplification cycles of 92 °C for 15 s and 60 °C for 1 min, as established by Ranade et al.^34^

### 2.7 Statistical analysis

PLINK software version 1.06 (https://zzz.bwh.harvard.edu/plink/ld) was used to analyze the allelic and genotype distributions, haplotype frequency, and Hardy-Weinberg equilibrium (HWE) within each genetic polymorphism. Genotype distribution was evaluated in co-dominant (AA *vs.* Aa *vs.* aa), dominant (AA *+* Aa *vs.* aa) and recessive (AA *vs.* Aa + aa) models.

To compare the genotype and allele distributions according to the dental pulp phenotype, chi-square tests and odds ratios (OR) with 95 % confidence intervals (CIs) were used.

Haplotype frequencies were compared using Fisher's exact test.

Narrowing of the radicular pulp space and pulp stones were grouped into ‘pulp calcification’ and ‘control (no calcification)’ for the statistical analysis.

Chi-square and t-tests were used to compare orthodontic variables according to the groups, such as age (start and end of treatment), duration of treatment, and skeletal facial profile.

All tests were performed with an alpha established in 5 % (0.05).

## 3 Results

The 'prevalence of patients with dental pulp calcifications after orthodontic treatment in at least one maxillary and/or mandibular first molar, in at least one maxillary first molar, and in at least one mandibular first molar were 42.4 % (n = 56), 37.9 % (n = 50), and 20.5 % (n = 27), respectively.

Among the 490 first molars evaluated, 248 presented dental pulp calcification.

The investigated genetic polymorphisms were in the HWE (chi‐squareHWE = 0.08 for rs2237051; chi‐squareHWE = 2.49 for rs4444903; chi‐squareHWE = 1.63 for rs2227983; chi‐squareHWE = 2.88 for rs763317; chi‐squareHWE = 0.19 for rs1800469; chi‐squareHWE = 2.04 for rs4803455; chi‐squareHWE = 0.29 for rs3087465; and chi‐squareHWE = 0.08 for rs764522).

Table 2 shows the orthodontic treatment variables (age at the start and end of treatment, treatment duration, and type of skeletal facial profile) of the pulp calcification and control groups (without calcification). These variables were not associated with pulp calcification (*P* > 0.05).

**Table 2:** Description of samples taking into consideration the orthodontic treatment variables: age at the start and end of treatment, treatment duration (in years) and type of facial profile.

Groups	Orthodontic treatment variables			
	Treatment duration (mean ± SD)	Age (start of treatment) (mean ± SD)	Age (end of treatment) (mean ± SD)	p-value
**Control (no calcification)**	1.84 (0.61)	14.34 (1.83)	16.09 (1.94)	>0.05
**Maxillary/Mandibular molars (with calcification)**	1.71 (1.31)	14.91 (2.60)	16.71 (2.66)	>0.05
**Maxillary molars (with calcification)**	1.74 (1.36)	15.00 (2.70)	16.82 (2.76)	>0.05
**Mandibular molars (with calcification)**	1.81 (0.68)	14.85 (2.41)	16.78 (2.22)	>0.05
Note: T-test was used to obtain p-value; mean and standard deviation in years				

Groups	Facial profile			
	Cl 1 (n-%)	Cl 2 (n-%)	Cl 3 (n-%)	p-value
**Control (no calcification)**	34 (44.7)	35 (46.1)	7 (9.2)	*reference*
**Maxillary/Mandibular molars (with calcification)**	26 (46.4)	29 (51.8)	1 (1.8)	>0.05
**Maxillary molars (with calcification)**	11 (44.7)	15 (55.6)	1 (3.7)	>0.05
**Mandibular molars (with calcification)**	24 (48.0)	26 (52.0)	0 (0.0)	>0.05
Note: Chi^2^ test was performed to compare and to obtain p-value. Cl means class.				

The genotype distributions in the co-dominant, dominant, and recessive models among the groups were analyzed and are presented in Table 3. There were no statistically significant differences between the groups (*P* > 0.05).

**Table 3:** Genotype distribution of *EGF* (rs2237051 and rs4444903), *EGFR* (rs2227983 and rs763317), *TGFβ1* (rs1800469 and rs4803455) and *TGFβR2* (rs3087465 and rs764522).

rs	Phenotype	Genotype (%)			p-value		
		AA	AG	GG	*Co-dominant*	*Dominant*	*Recessive*
rs2237051	Control (no calcification)	10 (7.57)	37 (28.03)	24 (18.18)	***reference***		
	Maxillary + mandibular 1st molar with calcification	11 (8.33)	25 (18.93)	17 (12.87)	0.614	0.839	0.327
	Maxillary 1st molar with calcification	10 (7.57)	22 (16.66)	15 (11.36)	0.598	0.831	0.314
	Mandibular 1st molar with calcification	5 (3.78)	12 (1.51)	9 (6.81)	0.894	0.850	0.725
rs4444903		**GG**	**GA**	**AA**	***Co-dominant***	***Dominant***	***Recessive***
	Control (no calcification)	7 (7.57)	38 (28.78)	28 (21.21)	***reference***		
	Maxillary + mandibular 1st molar with calcification	5 (3.78)	28 (21.21)	20 (15.15)	0.996	0.943	0.976
	Maxillary 1st molar with calcification	4 (3.03)	25 (18.93)	18 (13.63)	0.955	0.971	0.765
	Mandibular 1st molar with calcification	2 (1.51)	14 (10.60)	11 (8.33)	0.892	0.749	0.672
rs2227983		**CC**	**CA**	**AA**	***Co-dominant***	***Dominant***	***Recessive***
	Control (no calcification)	8 (6.06)	27 (20.45)	37 (28.03)	***reference***		
	Maxillary + mandibular 1st molar with calcification	4 (3.03)	17 (12.87)	33 (25.00)	0.524	0.277	0.483
	Maxillary 1st molar with calcification	4 (3.03)	14 (10.60)	30 (22.72)	0.466	0.218	0.721
	Mandibular 1st molar with calcification	1 (0.75)	12 (1.51)	15 (11.36)	0.365	0.810	0.223
rs763317		**GG**	**GA**	**AA**	***Co-dominant***	***Dominant***	***Recessive***
	Control (calcification)	11 (8.33)	43 (32.57)	18 (13.63)	***reference***		
	Maxillary + mandibular 1st molar with calcification	14 (10.60)	30 (22.72)	11 (8.33)	0.345	0.506	0.153
	Maxillary 1st molar with calcification	13 (9.84)	26 (19.69)	10 (7.57)	0.304	0.605	0.124
	Mandibular 1st molar with calcification	6 (4.54)	16 (12.12)	6 (4.54)	0.957	0.840	0.792
rs1800469		**AA**	**AG**	**GG**	***Co-dominant***	***Dominant***	***Recessive***
	Control (no calcification)	4 (3.03)	30 (22.72)	36 (27.27)	***reference***		
	Maxillary + mandibular 1st molar with calcification	4 (3.03)	21 (15.90)	30 (22.72)	0.844	0.729	0.723
	Maxillary 1st molar with calcification	4 (3.03)	19 (14.39)	26 (19.69)	0.789	0.962	0.517
	Mandibular 1st molar with calcification	11 (8.33)	13 (9.84)	13 (9.84)	0.610	0.584	0.518
rs4803455		**CC**	**CA**	**AA**	***Co-dominant***	***Dominant***	***Recessive***
	Control (no calcification)	12 (9.09)	38 (28.78)	18 (13.63)	***reference***		
	Maxillary + mandibular 1st molar with calcification	10 (7.57)	28 (21.21)	11 (8.33)	0.856	0.619	0.706
	Maxillary 1st molar with calcification	9 (6.81)	24 (18.18)	10 (7.57)	0.890	0.770	0653
	Mandibular 1st molar with calcification	5 (3.78)	13 (9.84)	5 (3.78)	0.887	0.705	0.687
rs3087465		**AA**	**AG**	**GG**	***Co-dominant***	***Dominant***	***Recessive***
	Control (no calcification)	7 (5.30)	37 (28.03)	28 (21.21)	***reference***		
	Maxillary + mandibular 1st molar with calcification	5 (3.78)	21 (15.90)	28 (21.21)	0.329	0.147	0.930
	Maxillary 1st molar with calcification	4 (3.03)	20 (15.15)	24 (18.18)	0.614	0.324	0.721
	Mandibular 1st molar with calcification	4 (3.03)	9 (6.81)	15 (11.36)	0.218	0.270	0.330
rs764522		**GG**	**GC**	**CC**	***Co-dominant***	***Dominant***	***Recessive***
	Control (no calcification)	2 (1.51)	16 (12.12)	53 (40.15)	***reference***		
	Maxillary + mandibular 1st molar with calcification	0 (0.00)	10 (7.57)	38 (28.78)	0.480	0.568	0.240
	Maxillary 1st molar with calcification	0 (0.00)	10 (7.57)	32 (24.24)	0.547	0.957	0.292
	Mandibular 1st molar with calcification	0 (0.00)	4 (3.03)	22 (16.66)	0.476	0.268	0.450

Table 4 shows the allelic distributions according to the groups. No alleles were associated with molar pulp calcification (*P* > 0.05).

**Table 4:** Allellic distribution of *EGF* (rs2237051 and rs4444903), *EGFR* (rs2227983 and rs763317), *TGFβ1* (rs1800469 and rs4803455) and *TGFβR2* (rs3087465 and rs764522).

Gene	Polymorphism	Phenotype	Minor Allele Frequency (%)	p-value	OR (95 % CI)
*EGF*	rs2237051	Control (no calcification)	40.14	***reference***	***reference***
		Maxillary + mandibular 1st molar with calcification	44.34	0.507	1.18 (0.71–1.98)
		Maxillary 1st molar with calcification	44.68	0.493	1.19 (0.71–2.01)
		Mandibular 1st molar with calcification	42.31	0.951	1.02 (0.54–1.89)
	rs4444903	Control (no calcification)	35.62	***reference***	***reference***
		Maxillary + mandibular 1st molar with calcification	35.85	0.969	1.01 (0.60–1.70)
		Maxillary 1st molar with calcification	35.11	0.876	0.95 (0.56–1.63)
		Mandibular 1st molar with calcification	33.33	0.680	0.87 (0.46–1.65)
*EGFR*	rs2227983	Control (no calcification)	29.86	***reference***	***reference***
		Maxillary + mandibular 1st molar with calcification	23.15	0.234	0.70 (0.40–1.25)
		Maxillary 1st molar with calcification	22.92	0.253	0.71 (0.39–1.27)
		Mandibular 1st molar with calcification	25	0.704	0.87 (0.44–1.73)
	rs763317	Control (no calcification)	45.14	***reference***	***reference***
		Maxillary + mandibular 1st molar with calcification	52.73	0.230	1.35 (0.82–2.23)
		Maxillary 1st molar with calcification	53.06	0.241	1.35 (0.81–2.24)
		Mandibular 1st molar with calcification	50	0.789	1.08 (0.59–1.96)
*TGFβ1*	rs1800469	Control (no calcification)	27.14	***reference***	***reference***
		Maxillary + mandibular 1st molar with calcification	26.36	0.890	0.96 (0.54–1.69)
		Maxillary 1st molar with calcification	27.55	0.829	1.06 (0.60–1.88)
		Mandibular 1st molar with calcification	27.78	0.854	1.06 (0.54–2.09)
	rs4803455	Control (no calcification)	45.59	***reference***	***reference***
		Maxillary + mandibular 1st molar with calcification	48.98	0.608	1.14 (0.68–1.92)
		Maxillary 1st molar with calcification	48.84	0.669	1.12 (0.65–1.91)
		Mandibular 1st molar with calcification	50	0.650	1.16 (0.60–2.21)
*TGFβR2*	rs3087465	Control (no calcification)	35.42	***reference***	***reference***
		Maxillary + mandibular 1st molar with calcification	28.70	0.260	0.73 (0.42–1.25)
		Maxillary 1st molar with calcification	29.17	0.37	0.77 (0.44–1.34)
		Mandibular 1st molar with calcification	30.36	0.692	0.87 (0.46–1.67)
	rs764522	Control (no calcification)	14.08	***reference***	***reference***
		Maxillary + mandibular 1st molar with calcification	10.42	0.403	0.70 (0.31–1.59)
		Maxillary 1st molar with calcification	11.90	0.81	0.90 (0.40–2.03)
		Mandibular 1st molar with calcification	76.92	0.22	0.51 (0.17–1.54)

Haplotypes frequency comparisons between the eight genetic polymorphisms were also performed and showed no statistically significant differences (*P* > 0.05).

## 4 Discussion

Formation of pulp calcifications is a common condition associated with many factors, triggers,^14^ and genetic backgrounds.^15^ Individual genetic predispositions might play a role in susceptibility to the formation of dental pulp calcifications.^8^^,^^26^ Orthodontic movement acts as a stress factor in the pulp and may generate inflammation at various levels.^13^ Therefore, dental pulp calcification may be triggered by orthodontic forces.^16^

Generally, dental pulp calcification poses no risk or damage to normal pulp physiology. However, pulp calcification may become a big concern if a root canal treatment is required.^35^ The accessibility of endodontic instruments to reach canal patency may be compromised and sometimes unfeasible^36^; dental pulp calcification can cause the obliteration or change the anatomy of endodontic access and root canal course, occlude the cervical entry of the root canals, and act as a physical barrier to endodontic file movement.^37^ Consequently, the root canal treatment outcome may be influenced by the increased risk of complications such as pulp chamber and root perforations, dental hard tissue loss, instrument fracture^38^^,^ and root canal working length loss.^36^^,^^37^

This study is the first to assess whether genetic polymorphisms in growth factor-encoding genes *EGF* (rs2237051 and rs4444903), *EGFR* (rs2227983 and rs763317), *TGFβ1* (rs1800469 and rs4803455) and *TGFβR2* (rs3087465 and rs764522) are associated with the risk of dental pulp calcification in orthodontic patients. The null hypothesis was accepted, indicating no statistical association between the selected genetic polymorphisms and occurrence of dental pulp calcification. The selection of the studied genetic polymorphisms was based in their potential clinical relevance and impact in the encoding protein.

Two well-known genetic polymorphisms in *TGFβ1* were selected and explored in this study. Some polymorphisms have been described in the coding and regulatory sequences of the *TGFβ1* gene, including the −509C/T functional promoter polymorphism (rs1800469) involving a cytosine-to-thymine transition. The −509C/T functional promoter polymorphism within the *TGFβ1* gene has been assessed in our study due its potential clinical relevance. This polymorphism has been report as regulating expression levels of TGFβ1 protein.^39^ A number of studies have attempted to investigate whether the genetic polymorphisms in *TGFβ1* change TGFβ1 expression.^40^^,^^41^ Another genetic polymorphism selected in this study is rs4803455 involving a cytosine-to-adenine transition, which is located in intron 2 of *TGFβ1*.^39^ This polymorphisms was associated with some clinical conditions, including in a systematic review.^42^ Although both genetic variations (rs1800469 and rs4803455) with known roles were hypothesized as potential candidates for pulp calcification, none of them were associated with the phenotype in our investigation.

Different pathways, related to proteins and extracellular organic matrix mineralization, and growth factors are involved in dentinogenesis. TGFβ signaling has been implicated in dentin formation and repair.^21^ TGFβ1 is a multifunctional cytokine that plays a crucial role in various cellular processes, including cell growth, differentiation, and immune response.^43^ Some previous studies associated calcifications with the TGFβ1 signaling pathway. Ahn et al.^21^ previously analyzed odontoblast-specific *Tgfbr2* conditional knockout mice. Their findings indicate that a decrease in the predentin layer caused by disrupted TGFβ signaling may promote matrix production in odontoblasts, resulting in osteodentin or pulpal obliteration depending on the responsiveness of odontoblasts to TGFβ signaling. Hu et al.^44^ showed the role of TGFβ1 in heart valve calcification induced by abnormal mechanical stimulation. Interestingly, previous studies have reported an association between coronary atherosclerosis and pulp calcification.^45^^,^^46^ Patients with connective tissue disorders, such as Marfan syndrome and several types of Loeys-Dietz syndrome, also exhibit calcification in tortuous vessels. These genetic aneurysmal conditions share an increased activity in the TGFβ1 signaling pathway, and it has been proposed that the calcification seen in these vessels is subsequent to the aberrant remodeling of the vessel wall.^47^^,^^48^ Elevated TGFβ1 levels are also frequently observed in patients with chronic kidney disease. Moreover, TGFβ1 contributes to the development of medial vascular calcification during hyperphosphatemia, a pathological process promoted by osteo-/chondrogenic transdifferentiation of vascular smooth muscle cells.^49^

TGFβ signals via two receptor serine/threonine protein kinases, TGFβR1 and TGFβR2. TGFβR2 is a constitutively active kinase that, in the presence of ligand, phosphorylates specific serine residues in TGFβR1, mediating its activation. Whereas, TGFβR1 alone is incapable of ligand binding, being activated only after the formation of the TGFβ/TGFβR2 complex.^50^ Several studies have found an association between two promoter genetic polymorphisms rs764522 (−1444C/G) and rs3087465 (−834A/G) in *TGFβR2* and some diseases, such as abdominal aortic aneurysm,^51^ and end-stage renal disease.^52^ However, in the present study, these polymorphisms were not associated with dental pulp calcification.

Regarding the *EGF* gene, two genetic polymorphisms were evaluated, rs2237051 and rs4444903. *EGF* rs2237051 is a non-synonymous polymorphism in the coding region of *EGF* that causes a change from isoleucine to methionine at amino acid position 708. The rs2237051 polymorphism have been associated with several types of tumors, inflammatory diseases, and also dental phenotypes such as generalized aggressive periodontitis.^53^ The rs4444903 polymorphism is located in the *EGF* 5′ untranslated region, contains a base change from guanine to adenine. This polymorphism is one of the most important and most studied variations in the *EGF* gene. Emerging evidence suggests that EGF enhances mineralization during the differentiation of bone marrow-derived mesenchymal stem cells.^54^

*EGFR* encodes the epidermal growth factor receptor, which is a transmembrane tyrosine kinase receptor that activates intracellular signaling pathways.^55^ Several genetic polymorphisms are located in the *EGFR* gene, including rs2227983 and rs763317. The polymorphism rs2227983 includes a guanine-to-adenine transition, leading to an arginine to lysine substitution. This missense variant leads to an attenuation in ligand binding and growth stimulation.^56^^,^^57^ The rs763317 polymorphism is located in the first intron of *EGFR* and was associated with some different phenotypes. *EGFR* polymorphisms lead to overexpression of the EGFR protein and have been associated with tumors.^58^

This study has some limitations. Although some of the orthodontic variables evaluated (e.g., age of patients, treatment duration, and type of malocclusion) were not associated with pulp calcification, other orthodontic variables, such as orthodontic force, could also influence the phenotype. A varied degrees of dental pulp inflammation could be provoked by this stress factor, that is also closely related to pulp calcification.^13^ However, as this variable is difficult to measure, it was not included in the study design. Differences in fixed orthodontic treatments may also contribute to the risk of dental pulp calcification. Another limitation of this study was the use of OPG to identify pulp calcifications. Although cone beam computed tomography imaging and micro-computed tomographic technology have arisen as gold standard for the study of dental hard tissues and root canals anatomy,^59^ the literature indicates that panoramic radiographs have been the most widely used.^7^ This examination allows the evaluation of all teeth simultaneously at the same time with a single exposure, uses minimal ionizing radiation, and is an integral part of dental checkups and orthodontic treatments. However, it also has some disadvantages, like limited resolution, the tendency to underreport calcifications (<200 μm), and structure overlap.^7^^,^^8^ In this study, the evaluation of pulp calcification was made through the analysis of panoramic radiographs since these exams are taken as follow-up images for the orthodontic treatment. Therefore, no additional image exams were performed in order to protect the patients from additional radiation exposure. Owing to the limitations of two-dimensional examinations, this study grouped pulp stones and narrowing of the radicular pulp space as a single phenotype. Although the absence of stratification of the pulp calcification phenotypes could also be a limitation, the hypothesis is that *EGF, EGFR, TGFβ1,* and *TGFβR2* act in both phenotypes in a similar way.

This study was also limited to the polymorphisms analyzed, which may have restricted or reduced phenotypic expression. In this study, genetic polymorphisms in *EGF, EGFR, TGFβ1*, and *TGFβR2* were not statistically associated with pulp calcifications. This suggests that rs2237051, rs4444903, rs2227983, rs763317, rs1800469, rs4803455, rs3087465, and rs764522 may not be the actual pathway in which these genes are acting in the onset of calcification and they are not genetic biomarkers of dental pulp calcification in orthodontic patients, even though these growth factors are promising candidate genes. Therefore, it is possible that other genetic polymorphisms in these genes could be involved in dental pulp calcification, once these genetic polymorphisms do not cover the whole genes.

This is the first study to investigate *EGF, EGFR, TGFβ1*, and *TGFβR2* genes and pulp calcification, bringing novel data about the role of genetic polymorphisms in growth factors and the susceptibility of orthodontic patients to develop pulp calcification. A strength of this study is that the sample analyzed in this study had similar socioeconomic, ethnic, and cultural backgrounds.

Understanding the hereditary contribution to the risk of pulp calcifications and identifying the genes that contribute to the pathogenesis of such conditions may be useful for risk assessment, allowing a more personalized orthodontic treatment plan. Therefore, similar studies should be conducted in the future.

## 5 Conclusion

Genetic polymorphisms in *EGF, EGFR, TGFβ1,* and *TGFβR2* were not associated with pulp calcifications in orthodontic patients. Further studies on different populations and other genetic polymorphisms of the evaluated genes are required.

## Funding

This study was financed in part by the 10.13039/501100002322Coordenação de Aperfeiçoamento de Pessoal de Nível Superior – Brasil (CAPES) – Finance Code 001 and Alexander-von-Humboldt-Foundation.

## Declaration of competing interest

The authors declare the following financial interests/personal relationships which may be considered as potential competing interests: Erika Calvano Kuchler reports was provided by University of Bonn. Erika Calvano Kuchler reports a relationship with University of Bonn that includes: employment. If there are other authors, they declare that they have no known competing financial interests or personal relationships that could have appeared to influence the work reported in this paper.
